# Ameliorating effect of *Allium Sativum* on high-fat diet induced fatty liver in albino rats

**DOI:** 10.12669/pjms.322.9025

**Published:** 2016

**Authors:** Aisha Qamar, Ambreen Usmani, Humera Waqar, Asma Siddiqui, Hemant Kumar

**Affiliations:** 1 Dr. Ayesha Qamar, M Phil, Department of Anatomy, Bahria University Medical and Dental College, Karachi – Pakistan; 2 Dr. Ambreen Usmani, M Phil, Department of Anatomy, Bahria University Medical and Dental College, Karachi – Pakistan; 3 Dr. Humera Waqar, MBBS, Department of Anatomy, Bahria University Medical and Dental College, Karachi – Pakistan; 4 Dr. Asma Siddiqui, M Phil, Department of Anatomy, Bahria University Medical and Dental College, Karachi – Pakistan; 5 Dr. Hemant Kumar, M Phil, Department of Anatomy, Hamdard University, Karachi – Pakistan

**Keywords:** High-fat diet, Fatty liver, Garlic

## Abstract

**Objective:**

To assess the hepatoprotective effect provided by fresh garlic on fatty liver induced by high-fat diet.

**Methods:**

This experimental study was carried out at BMSI, JPMC from October to November 2008. Thirty adult albino rats, 200-240 gram weight, were divided into three groups. Group A received control diet, Group B received high-fat diet (20 mg butter/100 gm diet) and Group C received high-fat diet with fresh garlic (20 mg butter with 6 gm fresh garlic/100 gm diet). The groups were further divided on the basis of duration of treatment, four weeks and eight weeks respectively. The rats were sacrificed, liver removed, weighed and relative liver weight calculated. Hepatic tissue was processed and tissue slides stained with haematoxylin and eosin.

**Results:**

There was significant increase in relative liver weight in group B animals as compared to the control animals, which decreased significantly in group C. Haematoxylin and eosin stained sections revealed ballooned hepatocytes having vesicular appearance with pyknotic nuclei in high-fat group which were preserved to a great extent in group C animals.

**Conclusion:**

This study has shown that use of fresh garlic along with high-fat diet prevents its damaging effects on liver to a great extent.

## INTRODUCTION

Non-alcoholic fatty liver disease (NAFLD) refers to a broad spectrum of pathological conditions affecting liver ranging from most common, reversible form, fatty liver to nonalcoholic steatohepatitis (NASH), to cirrhosis which is an advanced, irreversible scarring of liver as a result of chronic inflammation.^1^ The pathogenesis of NAFLD suggests a “multi-hit” model, in which metabolic derangement of free fatty acids is regarded as the “first hit”, leading to insulin-resistance and deposition of fat in the liver. Inflammatory response, oxidative stress, apoptosis, and autophagy serve as “following-hits” that leads to a cascade of inflammation, progressively damaging the liver.^2^ Without proper management, NAFLD may advance to liver failure and hepatocellular carcinoma.^3^

Evidence from various studies has established that high-fat westernized diets may not only induce hypercholesterolemia but also lead to collateral features of metabolic syndrome, such as decreased HDL levels, hypertriglyceridemia, hyperinsulinemia, insulin resistance, abdominal obesity, and nonalcoholic fatty liver disease.^4^

Commercial butter contains 80% butter fat and 15% water, with a high proportion of saturated fat. Saturated fat consists of triglycerides containing saturated fatty acids such as palmitic, oleic, myristic, and stearic acids. Fats rich in saturated fatty acids can result in the elevation of plasma total and lipoprotein cholesterol.^5^

Extensive clinical and scientific studies on obesity have focused on the search for food ingredients that have the capability to stimulate energy expenditure. Garlic (*Allium sativum*) has been used as a herbal remedy for a long time. Many studies have shown beneficial effects of garlic, which include hypocholesterolemic,^6^ hypoglycemic,^7^ anticancer^8^ and antioxidant effects.^9^ The extracts of garlic contain various biologically active compounds such as alliin, allicin, ally methanethiosulfinate, ajoene, diallyl disulfide, diallyl trisulfide, and S-allyl cysteine.^10^ The hepato-protective effects of garlic extract have been observed in acetaminophen^11^ and a carbon tetrachloride-induced acute liver injury.^12^ However, mechanisms underlying the protective effects of garlic in NAFLD are required to be identified. In this study, we investigated that whether administration of fresh crushed garlic along with high-fat diet to orally fed rat model effectively reduced NAFLD-induced hepatic injury.

## METHODS

This study was conducted in the department of Anatomy, Basic Medical Sciences Institute (BMSI), Jinnah Postgraduate Medical Center (JPMC), Karachi for 8 weeks from October to November 2008.

Thirty adult, healthy albino rats, 90-120 days old, weighing from 200-240 gram were taken for this prospective experimental study. The animals were kept under observation for one week prior to the commencement of the study, for the assessment of their health status and amount of diet intake. The animals were divided into three groups according to diet plan (Table-I). Each group was further divided into two subgroups A1, A2; B1, B2 and C1, C2 based on the duration of treatment i.e. 4 and 8 weeks respectively. Each subgroup comprised of 5 animals.

**Table-I:** Calories of diet/day for experimental albino rats.

Items of Diet	Quantity	Normal Diet				High-Fat Diet			

		Energy	Fat (G)	Protein (G)	Carb (G)	Energy	Fat (G)	Protein (G)	Carb (G)
Wheat flour (G)	11.2	38.08	0.22	1.34	7.84	38.08	0.22	1.34	7.84
Chick peas (G)	2	4.93	0.07	0.26	0.82	4.93	0.07	0.26	0.82
Milk powder (G)	2.8	14.05	0.72	0.7	1.62	14.05	0.72	0.7	1.62
Butter (G)	3.2	-	-	-	-	22.4	2.62	-	-
Drinking water	Ad Libitum	-	-	-	-	-	-	-	-
Final Energy	-	57.06*, 3.56**	1.01	2.3	10.28	79.46*, 4.13**	3.63	2.3	10.28

* Key: Kcal,

** Kcal/G of diet, Carb = Carbohydrate

***Group A:*** Comprising of 10 animals served as control. They received normal diet.^13^

***Group B:*** Comprising of 10 animals received high-fat diet (20 mg butter/100 gm diet).^14^

***Group C:*** Comprising of 10 animals received high-fat diet along with fresh crushed garlic (20 mg butter with 6 gm fresh garlic/100 gm diet).^15^

The animals were weighed and kept in cages, with twelve hour light and dark cycle, under laboratory environment. Calculated amount of food was given (Table-I). Animals were sacrificed at the end of study.

A midline, longitudinal incision was given in the abdomen. Liver was exposed and its absolute weight was recorded on Sartorius balance. The relative weight of liver was calculated.^16^ After weighing, liver was washed with normal saline and block of tissue comprising of 2mm was fixed in buffered neutral formalin for 24 hours. Then it was processed in alcohol and embedded in paraffin.^17^ Four micron (4µm) thick sections were cut on rotary microtome. Paraffin embedded tissue was stained with Haematoxylin and Eosin^17^ to study general architecture of liver tissue under oil immersion lens. The results were evaluated by student “t” test. P-value was considered for significant differences.

## RESULTS

The mean values of relative liver weight (gram) in control subgroups A-1 and A-2 were 2.11±0.10 and 2.49±0.12 respectively while in butter treated subgroups B-1 and B-2 were 3.06±0.13 and 3.51±0.13 respectively. The data showed that there was highly significant increase (P<0.001) in relative liver weight in subgroups B-1 and B-2 when compared to corresponding control subgroups A-1 and A-2 (Table-II).

**Table-II:** Mean relative liver weight (gm/100gm) in different groups of Albino rat.

	4 Weeks	8 Weeks
Group A	2.11	2.49
Group B	3.06	3.51
Group C	2.46	2.86

The mean values of relative liver weight (gram) in butter with garlic treated subgroups C-1 and C-2 were 2.46±0.23 and 2.86±0.13 respectively. This data showed that there was significant decrease (P<0.05) in relative liver weight in subgroup C-1, whereas moderately significant decrease (P<0.005) in subgroup C-2 when compared with butter treated subgroups B-1 and B-2 respectively. The data also showed significant increase (P<0.05) in subgroup C-1, whereas insignificant increase (P>0.05) in subgroup C-2 when compared with corresponding control subgroups A-1 and A-2 (Table-II).

**Fig.1:**
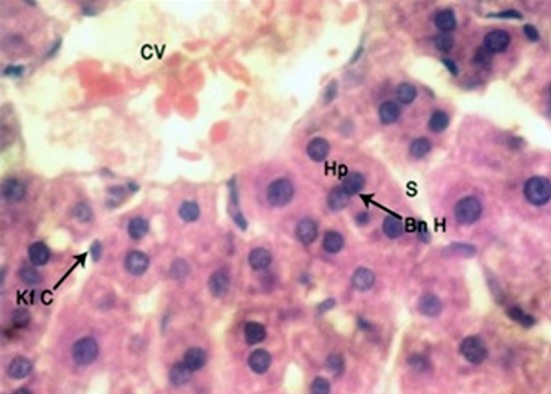
H and E stained, 4μm thick section of rat liver showing hepatocytes (Hp), central vein (CV), binucleate hepatocytes (Bn H), regular sinusoids (S) and Kupffer cells (Kf C) (Photomicrograph x 1000).

The H and E stained sections in subgroups A-1 and A-2 showed organized plates of polygonal hepatocytes, radiating from the central vein to the periphery of lobule. Hepatocytes showed distinct boundaries with uniformly distributed, granular, eosinophilic cytoplasm. Nuclei were round and located centrally within hepatocytes, showing even distribution of chromatin. Few binucleate cells were also seen. Sinusoids showed variation in caliber, but lining was smooth and endothelial cells were visible. Fixed monocytes (Kupffer cells) were present in the lining of sinusoids (Fig.1).

**Fig.2:**
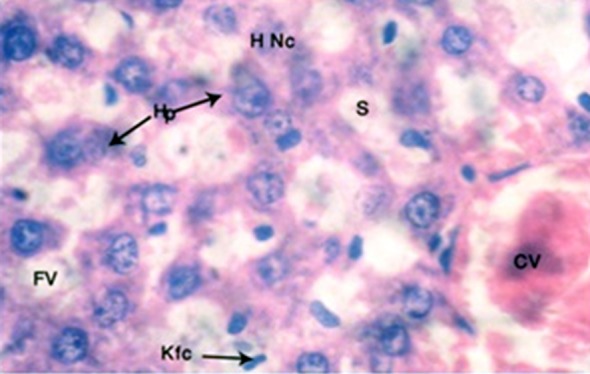
H and E stained, 4μm thick section of rat liver showing central vein (CV) slightly enlarged hepatocytes (Hp) showing small vaculoes (FV), nocrosed hepatocytes (H Nc), dilated sinusoids (S)and prominent Kupffer cells (Kf C) after 4 weeks treatment with butter (Photomicrograph x 1000).

The H and E stained sections in subgroup B-1 showed disruption of lobular architecture of hepatocytes. Hepatocytes appeared swollen, with empty spaces or vacuoles due to accumulation of fat droplets within them. Hepatocyte nuclei showed lightly stained, uneven chromatin pattern and pyknosis in many cells. Some hepatocytes showed necrosis. Binucleate cells were also frequent. The lumen of central veins was wide with congestion. The sinusoids were dilated as compared to control animals, with congestion. Kupffer cells were numerous (Fig.2).

**Fig.3:**
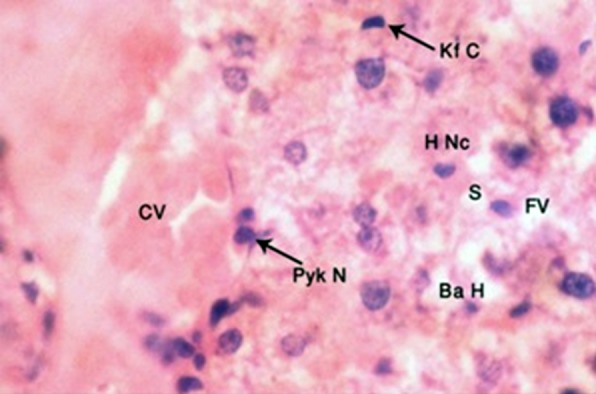
H and E stained, 4μm thick section of rat liver showing central vein (CV), ballooning of hepatocytes (Ba H), pyknotic nuclei (Pyk N), necrosed hepatocytes (H Nc), Kupffer cells (Kf C) after 8 weeks treatment with butter (Photomicrograph x 1000).

In subgroup B-2, hepatic lobular architecture was highly disorganized. Central vein was markedly dilated and congested, with distortion of walls and hemorrhage. Hepatocytes were markedly increased in size, vesicular in appearance due to accumulation of lipid droplets of varying size. Ballooned hepatocytes with indistinct cell membranes and large empty spaces due to fat vacuoles were also visible. Nuclei were small, shrunken and disintegrated. The number of necrosed cells and disintegrated nuclei were also increased as compared to subgroup B-1. Sinusoids were dilated at places, whereas at other places they were shrunken. Kupffer cells were prominent (Fig.3).

**Fig.4:**
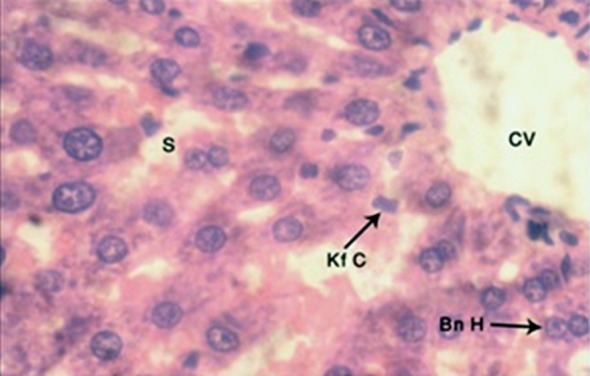
H and E stained, 4μm thick section of rat liver showing central vein (CV), binucleate hepatocytes (Bn H) and Kupffer cells (Kf C) in the lining of normal sinusoids (S) after 4 weeks treatment with butter and garlic (Photomicrograph x 1000).

The H and E stained sections in subgroup C-1 showed disruption of hepatic lobular architecture. There was no dilatation or congestion of central vein, with distinct endothelial lining. Hepatic sinusoids appeared normal with even caliber. Hepatocytes were normal in size similar to control, with distinct boundaries and preserved nuclei. Some of the cells showed pyknotic nuclei. Chromatin pattern was normal with distinct nucleoli. Binucleate cells were frequent. Kupffer cells were prominent (Fig.4).

In subgroup C-2, hepatic lobular architecture was preserved. Central vein showed distinct endothelial lining similar to control. Sinusoids were normal with even caliber (Fig.1). Hepatocytes were normal in shape and size similar to control.

## DISCUSSION

Hepatic lipid metabolism is a balance between lipid import and export from the liver.^1^ The accumulation of triglycerides in the liver occurs in response to an increased flux of fatty acids, from dietary sources, from adipose tissue or from enhanced synthesis of fatty acids in obesity.^12^

Despite serious attempts made by researchers, few choices are available to reverse or even retard progression of NAFLD.^3^ To date, weight loss is a high priority to improve liver injury induced by NAFLD.^18^ The role of natural food items which can be used daily has been searched for. Garlic has been drawing experimental and clinical attention for a long time due to its promising lipid-lowering effects. So this histological study was undertaken to evaluate the role of fresh crushed garlic on high fat diet-induced fatty liver.

The increase in relative liver weight in butter treated subgroups B1 and B2 animals was due to fatty change, causing hypertrophy of hepatocytes due to accumulation of fat, as described by Kumar et al.^19^ who described similar findings in fatty change in liver. This finding was in accordance to D’souza et al.^1^ who also observed highly significant increase in relative liver weight in male Sprague-Dawley rats receiving high-fat (60% calories from fat) and corticosterone group.

The significant decrease in relative weight of liver in group C animals as compared to group B was due to protection provided by garlic administration. In these groups, there was less deposition of fat in liver and decreased size of hepatocytes. These findings were in accordance to Rajasree et al.^20^ who also observed significant reduction in liver weight in alcohol fed rats after simultaneous feeding of garlic protein as compared to alcohol fed controls. They described that this was due to reduced activity of hydroxyl methyl glutaryl Co-enzyme A reductase in liver and increased hepatic degradation of cholesterol to bile acids.

The histopathological findings of butter treated group B animals were in agreement with findings of El Kot et al.^21^ The disruption of hepatic lobular architecture was directly related to period of exposure to high quantity of butter, which was obvious in changes in morphology between four and eight week butter treated animals. The liver showed congested and dilated central veins, uneven and congested sinusoids and swollen, as well as ballooned up hepatocytes. The changes were more marked in eight week subgroup as compared to four week butter-treated animals. Hepatocytes also showed multiple, small empty spaces depicting presence of microvesicular steatosis, which was similar to the findings of Zuniga et al.^22^ who also showed hepatocytes were filled with multilocular fat droplets of varying sizes due to lithogenic diet in mice. Some hepatocytes showed shrinkage of nuclei, with disruption of chromatin material (Fig.2 and 3). These findings were most likely due to influx of excessive saturated dietary fat, which could not be oxidized or leave the organ as enough apoproteins were not available, leading to excess deposition of triglycerides in hepatocytes, same as described by Tiniakos et al.^23^ Excess fats also caused damage to cell membranes by lipid peroxidation, leading to dilatation of veins. El Kot^21^ described similar findings showing hepatocyte degeneration, swelling and apoptosis by using sodium nitrate along with normal diet.

The observations of present study in group C1 (butter with garlic, four weeks) showed that protective effect of garlic reverted hepatic lobular architecture, but some hepatocytes showed vesicular cytoplasm with pyknotic nuclei, which was in agreement with Sharma et al.^24^ who also showed that after using low dose aged garlic extract in sodium nitrate-treated liver, some hepatocytes showed vacuolized cytoplasm and central vein appeared congested (Fig.4). The anti oxidative activities of garlic could be related to its contents of cysteine containing bioactive compounds, diallyl sulfur compounds and flavonoids, which are known to exert antioxidant effects. The size of hepatocytes significantly decreased similar to control in group C2. Metwally et al.^25^ also observed that liver restored most of its normal structure and was able to diminish fibrosis, congestion, inflammatory cells infiltration, centrilobular hepatocyte swelling and hepatocyte vacuolization in lead-induced hepatotoxicity after co treatment with Allium sativum.^25^

## CONCLUSION

From the results, it can be concluded that administration of fresh crushed garlic has beneficial effects in reducing high-fat diet induced fat accumulation in hepatocytes, and also preserves the normal morphology of liver.

## References

[1] D’souza AM, Beaudry JL, Szigiato AA, Trumble SJ, Snook LA, Bonen A (2012). Muscle Health Research Center and Physical Activity and Chronic Disease Consumption of a high-fat diet rapidly exacerbates the development of fatty liver disease that occurs with chronically elevated glucocorticoids. Am J Physiol Gastrointest Liver Physiol.

[2] Xiao J, Guo R, Fung M, Liong EC, Chang RCC, Ching Y (2013). Garlic-Derived S-Allylmercaptocysteine Ameliorates Nonalcoholic Fatty Liver Disease in a Rat Model through Inhibition of Apoptosis and Enhancing Autophagy. Evidence-Based Complementary and Alternative Medicine.

[3] Lin H, Chen C, Chen Y, Lin Y, Mersmann HJ, Ding S (2014). Enhanced Amelioration of High-Fat Diet-Induced Fatty Liver by Docosahexaenoic Acid and Lysine Supplementations. Bio Med Rese Int.

[4] Islam MS, Islam MK, Das SK (2014). Effects of butter and estrogen on lipid profile and histotexture of liver and skin in reference to the development of obesity in Swiss albino mice. Int J Innovation Applied Studies.

[5] Hartvigsen K, Binder CJ, Hansen LF (2007). A diet-induced hypercholesterolemic murine model to study atherogenesis without obesity and metabolic syndrome. Arterioscler Thromb Vasc Biol.

[6] Yeh YY, Liu L (2001). Cholesterol-lowering effect of garlic extracts and organosulfur compounds: human and animal studies. J Nutr.

[7] Jalal R, Bagheri SM, Moghimi A, Rasuli MB (2007). Hypoglycemic effect of aqueous shallot and garlic extracts in rats with fructose-induced insulin resistance. J Clin Biochem Nutr.

[8] Milner JA (2001). A historical perspective on garlic and cancer. J Nutr.

[9] Banerjee SK, Mukherjee PK, Maulik SK (2003). Garlic as an antioxidant: the good, the bad and the ugly. Phytother Res.

[10] Agarwal KC (1996). Therapeutic actions of garlic constituents. Med Res Rev.

[11] Sumioka Matsura T, Yamada K (2001). Therapeutic effect of S-allylmercaptocysteine on acetaminophen-induced liver injury in mice. Euro J Pharmacol.

[12] Xiao J, Liong EC, Ling MT, Ching YP, Fung ML, Tipoe GL (2012). S-allylmercaptocysteine reduces carbon tetrachloride-induced hepatic oxidative stress and necroinflammation via nuclear factor kappa B-dependent pathways in mice. Eur J Nutr.

[13] Mahan LK, Arlin MT (1992). Krause’s Food, Nutrition and Diet Therapy.

[14] Woods SC, Seeley RJ, Rushing PA, D’Alessio D, Tso P (2003). Controlled High-fat Diet Induces an Obese Syndrome in Rats. J Nutr.

[15] Qureshi AA, Abuirmeileh N, Din ZZ (1983). Inhibition of cholesterol and fatty acid biosynthesis in liver enzymes and chicken hepatocytes by polar fractions of garlic. Lipids.

[16] Sohrabi D, Alipour M, Mellati AA (2007). Effects of metronidazole on spermatogenesis, plasma gonadotrophins and testosterone in rats. Iranian J Reprod Med.

[17] Bancroft JD, Gamble M (2008). Theory and practical of Histological Techniques.

[18] Centis E, Marzocchi R, Domizio D, Ciaravella MF, Marchesini G (2010). The effect of lifestyle changes in non-alcoholicfatty liver disease. Digest Dis.

[19] Kumar V, Abbas AK, Fausto N, Mitchell RN (2007). Robbins Basic Pathology.

[20] Rajasree CR, Rajmohan T, Augusti KT (2009). Antiatherogenic and antiperoxidative effects of garlic and soy proteins in alcohol fed rats. Indian J Exp Biol.

[21] El-Kot AF, Abdel-Aziz AM, El-Latif AM, El-Gamel EM, Khalil AM (2012). Amelioration of Nitrate-induced hepatotoxicity by *Allium Sativum* in mice. J Med Sci.

[22] Zuniga S, Molina H, Azocar L, Amigo L, Nervi F, Pimentel F (2008). Ezitimibe prevents cholesterol gall stone formation in mice. Liver Int.

[23] Tiniakos DG, Vos MB, Brunt EM (2010). Nonalcoholic fatty liver disease: pathology and pathogenesis. Annu Rev Pathol.

[24] Sharma A, Sharma V, Kansal L (2010). Amelioration of lead induced hepatotoxicity by Allium sativum extracts in Swiss albino mice. Libyan J Med.

[25] Metwally MM, Hashem MA (2009). Protective role of garlic against cadmium toxicity in rats: Clinicopathological and histopathological studies Egypt. J Comp Path Clinic Path.

